# Unexplained dyspnea linked to mitochondrial myopathy following military deployment to Southwest Asia and Afghanistan

**DOI:** 10.14814/phy2.15520

**Published:** 2023-01-25

**Authors:** Claudia Daniela Onofrei, Eva Brigitte Gottschall, Lauren Zell‐Baran, Cecile Stephanie Rose, Richard Kraus, Kathy Pang, Silpa Dhoma Krefft

**Affiliations:** ^1^ Division of Pulmonary and Critical Care Medicine, Department of Medicine National Jewish Health Denver Colorado USA; ^2^ Division of Environmental and Occupational Health Sciences, Department of Medicine National Jewish Health Denver Colorado USA; ^3^ Division of Pulmonary and Critical Care Medicine, Department of Medicine University of Colorado Anschutz Medical Campus Colorado Aurora USA; ^4^ Department of Environmental and Occupational Health Colorado School of Public Health Colorado Aurora USA; ^5^ Division of Pulmonary and Critical Care Medicine, Department of Medicine Veterans Administration Eastern Colorado Health Care System Colorado Aurora USA

**Keywords:** Afghanistan, cardiopulmonary exercise testing, deployment, dyspnea, Iraq, mitochondrial myopathy

## Abstract

We identified a case of probable mitochondrial myopathy (MM) in a soldier with dyspnea and reduced exercise tolerance through cardiopulmonary exercise testing (CPET) following Southwest Asia (SWA) deployment. Muscle biopsy showed myopathic features. We compared demographic, occupational exposure, and clinical characteristics in symptomatic military deployers with and without probable MM diagnosed by CPET criteria. We evaluated 235 symptomatic military personnel who deployed to SWA and/or Afghanistan between 2010 and 2021. Of these, 168 underwent cycle ergometer maximal CPET with an indwelling arterial line. We defined probable MM based on five CPET criteria: arterial peak exercise lactate >12 mEq/L, anaerobic threshold (AT) ≤50%, maximum oxygen consumption (VO2_max_) <95% predicted, oxygen (O2) pulse percent predicted (pp) at least 10% lower than heart rate pp, and elevated ventilatory equivalent for O2 at end exercise (VE/VO2 ≥ 40). We characterized demographics, smoking status/pack‐years, spirometry, and deployment exposures, and used descriptive statistics to compare findings in those with and without probable MM. We found 9/168 (5.4%) deployers with probable MM. Compared to symptomatic deployers without probable MM, they were younger (*p* = 0.0025) and had lower mean BMI (*p* = 0.02). They had a higher mean forced expiratory volume (FEV1)_pp_ (*p* = 0.02) and mean arterial oxygen partial pressure (PaO2) at maximum exercise (*p* = 0.0003). We found no significant differences in smoking status, deployment frequency/duration, or inhalational exposures. Our findings suggest that mitochondrial myopathy may be a cause of dyspnea and reduced exercise tolerance in a subset of previously deployed military personnel. CPET with arterial line may assist with MM diagnosis and management.

## Abstract 2

After identifying a case of probable mitochondrial myopathy (MM) in a soldier with dyspnea and reduced exercise tolerance on cardiopulmonary exercise testing (CPET), we examined 168 CPETs and identified 9 cases of probable MM. Although there were no significant differences in smoking status, deployment frequency/duration, or inhalational exposures between those with and without probable MM, soldiers with MM were younger, had lower body mass index, higher forced expiratory volume in one second percent predicted values on spirometry and greater mean arterial oxygen partial pressure (PaO2) measurements at maximum exercise than dyspneic soldiers without probable MM. Cardiopulmonary exercise testing may aid in the diagnosis of mitochondrial myopathy in Southwest Asia‐deployed veterans with persistent, disabling and unexplained shortness of breath.

## 1 INTRODUCTION

Land‐based military personnel deployed to Southwest Asia (SWA) and Afghanistan since September 11, 2001, report exposure to a wide range of inhalational hazards such as desert dust, burn pit combustion products, diesel exhaust, and occupational vapors, dusts, gases, and fumes including those associated with combat duties (Zell‐Baran et al., 2019). A substantial number of soldiers experience persistent post‐deployment respiratory symptoms, primarily shortness of breath and decreased exercise tolerance. These symptoms have been linked to several respiratory diseases including asthma, bronchiolitis, tracheobronchomalacia, and rhinosinusitis (Gray et al., 2002; Krefft et al., 2020, 2021). Earlier deployments to SWA during the First Gulf War (1990–1991) were linked to respiratory hazards like those encountered during SWA deployments in the post‐9/11 era (National Academies of Sciences Engineering and Medicine, 2020). Previous studies have shown that conditions such as Gulf War Illness or Chronic Multi‐symptom Illness in First Gulf War veterans may be related to acquired mitochondrial dysfunction (Chen et al., 2017; Golomb, 2012; Koslik et al., 2014).

Mitochondria are key organelles in cellular energy production. Whether primary or secondary, mitochondrial dysfunction leads to loss of efficiency in the electron transport chain and reduction in synthesis of energy molecules such as adenosine tri‐phosphate (ATP) as well as defects in oxidative phosphorylation. While most cases of mitochondrial dysfunction have a subtle clinical presentation, in extreme cases it can lead to respiratory failure and persistent lactic acidosis. Both skeletal muscle and the myocardium can be affected by mitochondrial dysfunction through ATP starvation (Clay et al., 2001). However, diagnosis is often challenging, especially in early or subtle disease, and diagnostic tests can be inconclusive (Clay et al., 2001; Pfeffer & Chinnery, 2013).

In addition to heritable genetic diseases, mitochondrial dysfunction has been linked to a number of neurodegenerative conditions (e.g., Parkinson's disease, Huntington's disease, amyotrophic lateral sclerosis, and Alzheimer's disease; Johri & Beal, 2012). Prevalence in the general population has been estimated at greater than one in 5000 adults (Ng & Turnbull, 2016). More recently, de Boer et al implicated mitochondrial dysfunction as a possible contributor to post‐acute sequelae of SARS‐CoV‐2 infection (PASC) based on excessive lactate production and decreased fatty acid oxidation identified on cardiopulmonary exercise testing (CPET) in patients with PASC compared to a historical cohort of normal healthy controls (de Boer et al., 2022).

We evaluated a 26‐year‐old active‐duty soldier who reported profoundly decreased exercise capacity, leg fatigue and shortness of breath following military deployment in Iraq. His severe dyspnea persisted despite sustained strength and cardiovascular training. He had difficulty completing the Army‐mandated physical fitness test, despite having had no limitations prior to deployment. He also reported leg fatigue and leg weakness as limiting factors. Resting pulmonary function was normal, as were methacholine challenge and high‐resolution chest computed tomographic (HRCT) imaging. Metabolic cardiopulmonary exercise testing (CPET) showed reduced exercise tolerance with reduced oxygen consumption and low anaerobic threshold as well as an abnormally elevated blood lactate level. Muscle biopsy showed nonspecific myopathic features that included mild hypertrophy of skeletal muscle. While ragged red fibers considered pathognomonic for MM were not demonstrated, these abnormalities in addition to CPET findings of reduced exercise capacity, excessive lactate production, abnormally low oxygen pulse, and a hyperventilatory response supported a diagnosis of MM (Flaherty et al., 2001). Based on this unexpected clinical diagnosis, we explored the role of CPET as a tool to identify probable MM in a cohort of soldiers with persistent dyspnea and exercise limitation following deployment to Iraq and/or Afghanistan since 9/11/2001. Furthermore, we examined whether reported deployment respiratory hazardous exposures, smoking or other demographic variables were linked to CPET findings suggesting MM.

## 2 MATERIALS AND METHODS

### 2.1 Patient recruitment and clinical evaluation

We enrolled consecutive former and active‐duty military personnel who sought evaluation for persistent and often disabling respiratory symptoms at the National Jewish Health Center for Deployment‐Related Lung Disease following at least one deployment to SWA and/or Afghanistan between February 2010 and September 2021. All 235 symptomatic deployers underwent comprehensive clinical evaluation including detailed personal, smoking, family, and occupational history as well as a complete physical examination. Comprehensive medication histories were obtained and screened for use of statins, non‐steroidal anti‐inflammatory drugs (NSAIDs), fluoroquinolones and other medications that have been linked to mitochondrial dysfunction (De Vries et al., 2020). Findings and diagnoses were entered in a Research Electronic Data Capture (REDCap) database (Harris et al., 2009; Krefft et al., 2020, 2021).

### 2.2 Exposure assessment

All study subjects completed a detailed questionnaire that elicited information on military deployment‐specific exposures including frequency and intensity of exposure to particulate matter generated from burn pits, sandstorms, and diesel exhaust. We examined weighted respiratory hazards for participating deployers using a modification of a previously published exposure matrix (Zell‐Baran et al., 2019). Each participant's particulate matter inhalational exposure index was calculated using the following formula: (Months Deployed*Frequency of Exposure to Burn Pits in days/month) + (Months Deployed*Frequency of Exposure to Sandstorms in days/month) + (Months Deployed*Frequency of Exposure to Diesel Exhaust in days/month).

### 2.3 Pulmonary function testing

Participants underwent pulmonary function testing (PFTs) including pre‐ and post‐bronchodilator spirometry and lung volumes by body plethysmography. Spirometry reference values were obtained from the National Health and Nutrition Examination Survey III (Hankinson et al., 1999). Data were analyzed as percent predicted (pp) values based on age, gender, height, and race according to American Thoracic Society/European Respiratory Society (ATS/ERS) guidelines (Pellegrino et al., 2005).

### 2.4 Cardiopulmonary exercise testing

We obtained CPET on 181/235 symptomatic deployers when clinically indicated using calibrated bicycle ergometry in accordance with previously published guidelines. We excluded 13 CPETs from analysis based on suboptimal test quality, e.g., if a study was limited by non‐cardiopulmonary factors such as knee or back pain, inability to pedal consistently, or exercise for less than 2 min. After placing an indwelling arterial line, the patient was asked to sit on the bicycle ergometer and baseline measurements were obtained. Following 3 min of unloaded biking, work was added with a ramp protocol, with the goal to have the patient reach their maximal exercise capacity within 6 to 12 min. An exercise physiologist and respiratory therapist monitored electrocardiography and pulse oximetry continuously, while also obtaining blood pressure, heart rate and arterial blood gas measurements every 2 min. A metabolic cart (Ultima CPX cardiO2) was used to analyze ventilatory parameters and oxygen and carbon dioxide in expired air breath‐by‐breath. The following variables were averaged sequentially every 15 to 30 s: oxygen consumption (VO_2_), carbon dioxide production (VCO_2_), and expired minute ventilation (VE). At the end of the test, symptom severity was assessed using the standardized Borg scale for dyspnea and leg fatigue.(Crisafulli & Clini, 2010) Findings were interpreted based on published guidelines.(American Thoracic Society, 2003)

### 2.5 Case definitions for probable mitochondrial myopathy

Published studies describe CPET features of MM as including reduced maximal whole body oxygen consumption (VO2 max) with a hyperdynamic cardiovascular response. There also is an imbalance in peripheral oxygen extraction in conjunction with hyperdynamic oxygen delivery (Tarnopolsky & Raha, 2005). Other CPET hallmarks of MM include reduced anaerobic threshold, and early and persistent increase in blood lactate level throughout exercise. Ventilatory equivalent for oxygen (VE/VO2) is excessively high at peak exercise. Ventilatory reserve is usually reduced in intrinsic lung conditions but may be unaffected in myopathies (Flaherty et al., 2001; Riley et al., 2017). Using these published parameters, we defined probable MM based on the presence of all five of the following CPET criteria (Table 1): peak arterial exercise lactate >12 mEq/L; anaerobic threshold (AT) ≤50%; maximum oxygen consumption (VO2 max <95%, a measure of aerobic capacity); oxygen (O2) pulse percent predicted (pp) at least 10% lower than heart rate_pp_ (a measure of cardiovascular response to exercise); and elevated ventilatory equivalent for oxygen at end exercise (VE/VO2 ≥ 40 [a measure of hyper‐ventilatory response to exercise]; Flaherty et al., 2001; Riley et al., 2017).

**TABLE 1:** Comparison of aggregate means (ranges) between those with and without CPET diagnostic criteria for probable mitochondrial myopathy (MM)

CPET criteria for MM	CPET measurements means (ranges)		
	CPET variable	Probable MM *n* = 9	No MM *n* = 159
Peak arterial exercise lactate >12 mEq/L	Peak arterial exercise lactate	12.9 (12.1–13.9)	9.7 (4.3–17)
Anaerobic threshold (AT) ≤ 50%	Anaerobic threshold	30.4 (20–40)	44.8 (25–82)
Maximum oxygen consumption (VO2_max_) <95%	VO2_max_	70.7 (60–89)	88.1 (40–145)
Difference between O2 pulse_pp_ and HR_pp_ at end exercise at least −10%	O2 pulse_pp_ – HR_pp_ at end exercise	−26.7 (−15 – −42)	5.5 (−47–76)
Ventilatory equivalent for O2 at end exercise (VE/VO2) ≥ 40	VE/VO2	51.2 (40–58)	43.9 (30–72)

### 2.6 Statistical analysis

We compared demographic and clinical characteristics, pulmonary function, CPET measurements, and deployment exposures between those with and without probable myopathy using t‐tests for continuous variables (using the Satterthwaite result) and Fisher Exact tests for categorical variables. To meet test assumptions, several variables (number of deployments, total months deployed, and inhalational exposure index) were log or square root transformed. A Bonferroni correction was used to account for multiple comparisons, with *p* < 0.001 considered statistically significant. All analyses were performed using SAS 9.4.

## 3 RESULTS

Nine of 168 symptomatic patients who had previously deployed (5.4%), including the index case, met all five CPET criteria for probable MM. Table 1 shows aggregate means for each of the CPET variables between those with and without probable MM.

Table 2 compares demographic and exposure variables in deployers with and without probable MM. Those with probable MM were younger (mean age 32.0 vs. 41.1, *p* = 0.0025) and had lower mean BMI (27.4 kg/m^2^ vs. 30.6 kg/m Gray et al., 2002, *p* = 0.02). Most with probable MM (89% vs. 58%, *p* = 0.09) were never smokers. None with probable MM used medications linked to mitochondrial dysfunction. There were no significant differences in deployment frequency/duration or the inhalational exposure index between groups.

**TABLE 2:** Demographic and exposure variables in symptomatic post‐9/11 deployers with and without probable mitochondrial myopathy (MM)

	Total *n* ^a^ for each variable	Probable MM (mean ± SD) *n* = 9	No MM (mean ± SD) *n* = 159	*p*‐value*
Demographic characteristics				
Age (years)	168	32.0 ± 6.5	41.1 ± 9.4	0.0025
Male	168	9 (100%)	137 (86%)	0.61
Non‐Hispanic white race/ethnicity	168	8 (89%)	123 (77%)	0.69
Never smoking status	168	8 (89%)	92 (58%)	0.09
Smoking pack‐years	63	5.0 ± 0.0	6.9 ± 8.6	0.83
Body mass index (kg/m^2^)	167	27.4 ± 3.4	30.6 ± 4.9	0.02
Deployment exposure characteristics				
Number of deployments, median (range)	167	2 (1–4)	2 (1–11)	0.98**
Total months deployed	167	17.9 ± 9.5	22.6 ± 18.2	0.67**
Inhalational exposure index	160	651.6 ± 451.2	882.9 ± 790.4	0.49***

^a^ *n* = number of study participants in which data available for each variable.

* Values in bold were statistically significant (*p* < 0.002).

** *p*‐value from comparison of log‐transformed data to meet test assumptions.

*** *p*‐value from comparison of square root transformed data to meet test assumptions.

Similar to Flaherty et al, resting pulmonary physiologic assessment with spirometry in our study did not detect any significant differences between the MM and no MM groups (Flaherty et al., 2001). As indicated in Table 3, none of the soldiers with probable MM had airflow obstruction on spirometry, and there were no significant differences after correction for multiple comparisons in mean forced vital capacity (FVC)_pp_ (104.2 in MM vs. 92.4 in no MM, *p* = 0.02), forced expiratory volume (FEV1)_pp_ (104.2 in MM vs. 92.4 in no MM, *p* = 0.02), or FEV1/FVC ratio between groups (82.1% in MM vs. 80.5% in no MM, *p* = 0.41). We also found no between‐group differences in maximum voluntary ventilation (MVV). These findings support the typically limited utility of resting pulmonary function testing in the diagnosis of MM, although reduced MVV and FVC may be indicators of neuromuscular weakness, particularly in severe disease (Clay et al., 2001; Dandurand et al., 1995).

**TABLE 3:** Comparison of lung function and cardiopulmonary exercise testing variable means in symptomatic deployers with and without probable mitochondrial myopathy (MM)

	Total *n* ^a^ for each variable	Probable MM (mean ± SD) *n* = 9	No MM (mean ± SD) *n* = 159	*p*‐value*
Spirometry				
FVC_pp_	167	104.2 ± 11.7	92.4 ± 13.5	0.02
FVC (liters)	167	5.7 ± 0.7	4.6 ± 1.0	**0.0009**
FEV_1pp_	167	106.9 ± 14.3	92.8 ± 14.2	0.02
FEV1 (liters)	167	4.7 ± 0.5	3.7 ± 0.8	**0.0002**
FEV_1_/FVC	167	82.1 ± 5.3	80.5 ± 6.4	0.41
Cardiopulmonary exercise testing				
VO2 at rest (L/min)	168	0.4 ± 0.1	0.4 ± 0.1	0.53
VO2max (L/min)	168	2.2 ± 0.4	2.4 ± 0.6	0.35
VO2max_pp_	168	70.7 ± 9.9	88.1 ± 20.3	**0.0005**
Max work achieved (Watts)	168	216.4 ± 44.4	201.9 ± 51.6	0.37
Max work achieved_pp_	168	89.3 ± 14.0	96.3 ± 20.9	0.22
RER at VO2 max	168	1.3 ± 0.1	1.2 ± 0.1	0.004
Resting HR (bpm)	168	88.0 ± 21.4	82.2 ± 14	0.44
HR at max exercise (bpm)	168	185.9 ± 12.8	163.1 ± 15.8	**0.0005**
Max achieved HR_pp_	166	98.4 ± 6.2	90.9 ± 8.2	0.006
VO2/HR_pp_ (O_2_ pulse)	168	71.8 ± 8.1	96.8 ± 20.3	**<0.0001**
VE at rest (L/min)	168	11.7 ± 2.9	12.4 ± 3.2	0.49
VE_max_ (L/min)	168	118.4 ± 23.7	103.5 ± 27.1	0.10
VE_max_/MVV (%)	168	64.8 ± 16.3	68.2 ± 16.5	0.56
MVV rest (L/min)	168	187.1 ± 30.6	155.9 ± 39.2	0.02
MVV peak (L/min)	168	64.8 ± 16.3	68.2 ± 16.5	0.56
MVV_pp_	168	105.9 ± 17.4	100.2 ± 21.6	0.37
VD/VT rest	165	0.2 ± 0.1	0.3 ± 0.1	0.42
VD/VT peak exercise	164	0.1 ± 0.1	0.2 ± 0.1	0.20
SpO2 rest	168	96.1 ± 2.1	95.6 ± 1.9	0.52
SpO2 peak exercise	168	95.8 ± 2.4	94.2 ± 2.4	0.08
PaO2 rest (mmHg)	168	80.9 ± 8.4	78.6 ± 7.7	0.44
PaO2 at max exercise (mmHg)	168	90.5 ± 5.6	79.9 ± 8.3	**0.0003**
A‐a difference rest (mmHg)	166	1.6 ± 3.5	3.3 ± 5.2	0.19
A‐a difference at max exercise (mmHg)	165	8.6 ± 6.5	14.1 ± 6.8	0.03
PaCO2 at rest (mmHg)	168	32.8 ± 3.8	32.9 ± 3.9	0.92
PaCO2 peak exercise (mmHg)	168	26.7 ± 3.4	29.8 ± 3.6	0.03
PET O2 peak exercise	168	96.6 ± 3.8	92.5 ± 4.4	0.01
PET CO2 peak exercise	168	31.1 ± 5.9	32.2 ± 4.6	0.60
VE/VO2 start of exercise	168	29.0 ± 8.3	28.6 ± 4.2	0.88
VE/VO2 peak exercise	168	51.2 ± 5.8	43.9 ± 7.0	0.005
VE/VCO2 start exercise	168	33.9 ± 4.9	36.1 ± 5.0	0.23
VE/VCO2 peak exercise	168	38.7 ± 5.5	36.8 ± 5.3	0.33
Borg score for leg fatigue	161	8.4 ± 1.6	7.8 ± 2.0	0.30
Borg score for shortness of breath	161	8.6 ± 1.1	8.2 ± 1.8	0.40

Abbreviations: A‐a difference, alveolar‐arterial difference; FEV1_pp_, %predicted forced expiratory volume in the first second; FVC_pp_, %predicted forced vital capacity; HR_pp_, %predicted heart rate; MVV_pp_, %predicted maximal voluntary ventilation in 1 min; PaCO2, partial pressure of carbon dioxide; PaO2, partial arterial pressure of oxygen; PET CO2, end‐tidal partial pressure of carbon dioxide; PET O2, end‐tidal partial pressure of oxygen; RER at max, respiratory equivalent ratio at max exercise; SpO2, oxygen saturation; VD/VT, physiologic dead space/tidal volume; VE, minute ventilation; VE/VCO2, ventilatory equivalents for carbon dioxide; VE/VO2, ventilatory equivalents for oxygen; VEmax, maximum minute ventilation; VO2, oxygen consumption; VO2max_pp_, %predicted Liters of oxygen consumed in 1 min.

^a^ *n* = number of study participants in which data available for each variable.

* Values in bold were statistically significant (*p* < 0.001).

Table 3 summarizes relevant CPET parameters for the probable MM and non‐MM groups, all of whom sought evaluation at our Center due to unexplained dyspnea and/or decreased exercise tolerance. The mean maximal achieved VO2 (VO2_max_) for the probable myopathy group was abnormally low and lower than for the non‐MM group. Only one of the nine with probable MM achieved a normal VO2_max_ of >84% predicted. The MM group achieved a higher mean workload measured in watts than the non‐MM group. However, maximum workload_pp_, a better indicator of age‐adjusted workload achieved, was lower in the MM group (89.3 vs 96.3, *p* = 0.22).

The cardiovascular response showed a significantly lower mean O_2_ pulse at end exercise for those soldiers with probable myopathy versus those without, which may be indicative of abnormal oxygen delivery and/or oxygen utilization. The mean AT was lower in the probable MM patients and lactate levels were higher, consistent with an earlier transition to anaerobic metabolism due to impairment of peripheral oxygen extraction as occurs in mitochondrial myopathy.

The ventilatory response to exercise showed that patients in both groups had ventilatory reserve at end exercise, as they did not reach their ventilatory ceiling based on VE/MVV. The VE_max_pp was similar in both groups. Mean ventilatory equivalent for oxygen as measured by VE/VO2 was abnormally increased in both probable myopathy and non‐myopathy groups. However, it was higher in those with suspected MM, indicating a hyperventilatory response to exercise due to early transition to anaerobic metabolism.

Gas exchange parameters were normal in both patient groups, excluding a significant problem with oxygen delivery due to intrinsic lung disease with diffusion abnormalities at the alveolar level. Mean Borg scores for leg fatigue and breathlessness did not significantly differ between the probable MM and the non‐MM groups.

## 4 DISCUSSION

A sentinel case of probable mitochondrial myopathy based on clinical presentation, profound abnormalities on metabolic exercise testing, and an abnormal muscle biopsy prompted us to investigate whether other dyspneic patients in our cohort of previously deployed military personnel were similarly affected. Using cardiopulmonary exercise testing criteria adapted from the published literature, we identified nine patients with probable MM (Flaherty et al., 2001; Riley et al., 2017). Our findings suggest that MM may explain persistent dyspnea and exercise limitation in a subset of military personnel previously deployed to SWA and Afghanistan.

We found that previously deployed patients with probable MM were younger and had lower BMI measurements than those without MM, indicating that personal characteristics (e.g., older age and obesity) do not explain dyspnea in those with probable MM. These patients had reduced exercise capacity associated with a low anaerobic threshold, excessive lactate production, and cardiac performance characterized by a lack of heart rate reserve (HRR < 15 bpm in all but one patient) associated with a low oxygen pulse, the latter suggesting either a problem with oxygen extraction (as occurs in MM) or an oxygen delivery problem (as can be seen in cardiovascular disease). Given the young mean age, non‐smoking status, and typically high job activity requirements as active‐duty soldiers, deconditioning or a cardiovascular cause for the CPET abnormalities in the MM group is unlikely.

It can be difficult to distinguish how the pattern of CPET in MM differs from a cardiac limitation to exercise. While elevated end‐exercise lactate is a key parameter for identifying MM, determining whether low oxygen pulse is due to cardiac or MM limitation with CPET alone can be difficult without measurement of central or mixed venous blood samples to assess arterial–venous oxygen content difference C(a‐v)O2 or invasive cardiac output measurements using Swan‐Ganz catheterization. In the absence of these measurements, an echocardiogram and cardiovascular evaluation is helpful in making a distinction between cardiac or MM exercise limitation (Sietsema et al., 2020). All patients underwent assessment for cardiac risk factors, previous heart disease history, and electrocardiographic monitoring for ischemia during exercise, with results reviewed by a cardiologist. Three out of the nine patients with probable MM underwent echocardiogram and cardiovascular evaluation with a cardiologist that excluded a cardiac limitation to exercise.

Not surprisingly, we found no differences in any pulmonary function test parameters between those with and without probable MM. While evidence regarding the utility of PFTs in MM is inconclusive, previous work suggests that decreased MIP/MEP and MVV may be found in some cases of severe acquired and primary MM. We also found no differences in rates of other clinical diagnoses, including rhinosinusitis, asthma, and inducible laryngeal obstruction, in those with and without MM.

Mitochondrial dysfunction following military deployment has been linked to the unremitting multi‐system symptoms of Gulf War Illness (GWI), a condition that reportedly affects 25%–30% of Operation Desert Storm and Operation Desert Shield veterans (Chen et al., 2017; Koslik et al., 2014). While risk factors and causes for mitochondrial dysregulation in Gulf War veterans remain unknown, exposure to complex mixtures of airborne particulates and chemicals such as oil well fire smoke, depleted uranium (DU), carbamate and organophosphate pesticides, and pyridostygmine bromide nerve agent prophylactic pills, have been considered (Golomb, 2012; National Academies of Sciences E Medicine, 2018). During both the First Gulf War and more recent post‐9/11/2001‐era conflicts in SWA and Afghanistan, in‐theater environmental exposures were similar though difficult to reconstruct for individual military personnel deployed to these austere settings. Environmental similarities between the two conflicts are linked to geogenic and anthropogenic factors such as temperature extremes and high ambient dust levels from sandstorms, smoke from burn pits, and diesel fumes (National Academies of Sciences E, Medicine, 2020). Certain metals (lead, cadmium, mercury, manganese) that have been identified in air sampling in SWA locations can accumulate preferentially in the mitochondria via calcium transporters.(Engelbrecht et al., 2009) Although our study did not find differences between overall deployment exposure characteristics in those with and without MM, it is likely that variability in individual exposures and in host/genetic susceptibility and repair mechanisms confer differential risk for MM.

Our study has a number of strengths and limitations. First, CPET analysis provides a useful noninvasive diagnostic tool to assess mitochondrial dysfunction. An additional strength of our study is the use of multiple CPET variables which, when examined in aggregate, strengthen the ability to discriminate between a pattern of mitochondrial myopathy and disease processes that can show overlap, such as deconditioning and cardiovascular disease (Riley et al., 2017). Similar to other diseases with exercise‐related abnormalities, there likely is a spectrum of MM disease severity. Our case definition included those with a low normal VO2 max_pp_ and a low normal anaerobic threshold in order to identify mild or early cases of MM, but still required other rigorous CPET criteria for MM such as excessive lactate production, evidence of hyperdynamic cardiovascular response, and excessive ventilatory response to exercise.

Study limitations included small cohort size and lack of systematic evaluation with muscle biopsy, mitochondrial DNA analysis, and quantitative oxidative enzyme levels, in part related to the retrospective nature of this investigation.”. Although we did not have access to muscle biopsies in 8 of 9 who met our CPET criteria for probable MM, the histopathology of muscle biopsy in this setting may be inconclusive. A normal muscle biopsy does not exclude mitochondrial myopathy (Clay et al., 2001). Furthermore, muscle biopsy in a suspected case of MM requires special handling including immediate freezing of the tissue sample for biochemical analysis and electron microscopy evaluation to increase the diagnostic yield (Vogel, 2001). Expertise in specimen handling and interpretation is not widely available and requires involvement of a specialty center. Finally, our study cohort was not a random sample of veterans returning from military theater, instead including all those consecutively seeking clinical evaluation for persistent and disabling exertional respiratory symptoms.

## 5 CONCLUSIONS

Given the number of soldiers reporting both systemic and respiratory symptoms after deployment, future research should explore links between mitochondrial dysfunction and exercise limitation in post‐9/11 era SWA deployed personnel and those with GWI from the First Gulf War era. Research efforts that pair both CPET data and assessment of quantitative mitochondrial DNA lesions and dysfunction may inform understanding of disease pathogenesis and possible treatment strategies. In the interim, CPET may aid in the noninvasive diagnosis of mitochondrial myopathy in SWA deployed veterans with persistent, disabling and unexplained dyspnea.

## AUTHOR CONTRIBUTIONS

CDO, EBG, and CSR conceived of and designed the study. CDO, EBG, CSR, RK, and SDK took care of the patients. LZB and KP collected data. LZB performed data analysis. All authors interpreted the data. CDO, EBG, CSR, and SDK drafted the manuscript. All authors critically reviewed the manuscript.

## FUNDING INFORMATION

This work was supported by the Office of the Assistant Secretary of Defense for Health Affairs, through the Peer Reviewed Medical Research Program under Award No. W81XWH‐16‐2‐0018 as well as the Department of Veterans Affairs Clinical Sciences and Research Development Program under Award No. 1 IK2 CX001779‐01A1. Additional funding support was provided through the Sergeant Sullivan Fund at National Jewish Health.

## CONFLICT OF INTEREST

All authors report no conflicts of interest.

## ETHICS STATEMENT

The study was approved by the National Jewish Health/ Biomedical Research Alliance of New York Institutional Review Board HS‐2689 with all study participants providing consent to participate and publish research findings.

## DISCLOSURE STATEMENT

Opinions, interpretations, conclusions and recommendations are those of the authors and are not necessarily endorsed by the Department of Defense or the Department of Veterans Affairs.
